# Development of the Japanese Version of the Linguistic Inquiry and Word Count Dictionary 2015

**DOI:** 10.3389/fpsyg.2022.841534

**Published:** 2022-03-07

**Authors:** Tasuku Igarashi, Shimpei Okuda, Kazutoshi Sasahara

**Affiliations:** ^1^Graduate School of Education and Human Development, Nagoya University, Nagoya, Japan; ^2^Graduate School of Informatics, Nagoya University, Nagoya, Japan; ^3^School of Environment and Society, Tokyo Institute of Technology, Tokyo, Japan

**Keywords:** LIWC, natural language processing, Japanese text analysis, word count approach, psychometrics

## Abstract

The Linguistic Inquiry and Word Count Dictionary 2015 (LIWC2015) is a standard text analysis dictionary that quantifies the linguistic and psychometric properties of English words. A Japanese version of the LIWC2015 dictionary (J-LIWC2015) has been expected in the fields of natural language processing and cross-cultural research. This study aims to create the J-LIWC2015 through systematic investigations of the original dictionary and Japanese corpora. The entire LIWC2015 dictionary was initially subjected to human and machine translation into Japanese. After verifying the frequency of use of the words in large corpora, frequent words and phrases that are unique to Japanese were added to the dictionary, followed by recategorization by psychologists. The updated dictionary indicated good internal consistency, semantic equivalence with the original LIWC2015 dictionary, and good construct validity in each category. The evidence suggests that the J-LIWC2015 dictionary is a powerful research tool in computational social science to scrutinize the psychological processes behind Japanese texts and promote standardized cross-cultural investigations in combination with LIWC dictionaries in different languages.

## Introduction

Understanding how people feel and think in their daily lives is a primary objective of social science. In human society, language is an essential tool for thinking and communicating, thereby comprehending one’s own internal state and that of others. From personal diaries to public speeches, informal conversations to social media posts, words reflect the ruminations and emotions of one’s mind and heart. Although social scientists have found the systematic quantitative analysis of text-based data challenging because of its complexity, recent advancements in computational linguistics have made it possible to evaluate the psychological meanings of language use.

Linguistic Inquiry and Word Count (LIWC; pronounced “Luke;” Chung and Pennebaker, 2012) is a *de facto* standard analytic framework to quantify psychological constructs embedded in text data. LIWC is composed of computer software and a dictionary file. The software classifies each word in a given text into multiple linguistic/psychological categories based on the words included in the dictionary file (hereafter referred to as “target words”) and calculates the proportion of the words in each category to the total number of words in the entire text. The dictionary file defines target words as a collection of frequently used words, each of which is linked to specific categories, such as positive/negative emotions, cognitive and perceptual processes, and personal and social concerns. Although LIWC can be used for simple sentiment analysis to study affective states such as positive and negative, it is not just a sentiment dictionary but rather a general research tool for inferring more complicated psychological states from texts.

LIWC has been updated constantly since its initial release in the 1990s (Pennebaker and Francis, 1996). It has been applied in various research topics, including the variability of the spread of false news online according to emotional reactions (Vosoughi et al., 2018), analysis of brand/product preferences in news media (Humphreys and Wang, 2018), mood contagion from charismatic leaders to followers (Bono and Ilies, 2006), and language style matching and relationship stability in dyads (Ireland et al., 2011), to name a few (see Tausczik and Pennebaker, 2010 for a review). The LIWC dictionary was initially developed in English and translated into German, simplified Chinese, traditional Chinese, Spanish, Russian, Arabic, French, Italian, Portuguese, Serbian, Romanian, and Turkish. The availability of localized dictionaries has led LIWC to become a gold standard for cross-cultural psycholinguistic analysis. However, LIWC has not been translated into Japanese in a publicly available format.

The LIWC dictionary was constructed and validated in a standardized manner. First, the target words are collected from large corpora in different communication contexts and screened based on their frequency of use. Then, the associations between the target words and the pre-defined psychologically meaningful categories are determined and judged by psychologists. Linguistic categories, such as personal pronouns, are also allocated to some target words. Each target word corresponds to multiple categories, organized both horizontally and hierarchically. For example, the target word “beauty” is classified into four categories: “affect” (level 1), “positive emotion” (level 2 under the affect category), “perceptual process” (level 1), and “see” (level 2 under the perceptual process category). Some target words include a wildcard character (“*”) at word endings to match any word that starts with a particular string of characters (e.g., “enjoy*” matches “enjoy,” “enjoyable,” “enjoyed,” and “enjoyment”). The use of the wildcard expands the concise dictionary to cover a broad range of inflected forms of the target words. As of 2021, the newest dictionary was developed in 2015 (LIWC2015), which includes approximately 6,400 target words and more than 70 linguistic/psychological categories.

The current research aims to develop a Japanese version of the LIWC2015 dictionary (J-LIWC2015). Several studies have attempted this recently (Nasugawa et al., 2016; Yamamoto et al., 2016; Shibata, 2018; Tomihira et al., 2018). However, these studies mainly used a machine translation technique to create a Japanese dictionary from the older versions of the original dictionaries (LIWC2001 and 2007), and the developed dictionaries are not open to the public. The translation of every single target word and its link to psychometric properties have also not been thoroughly reviewed by experts. To date, no standardized Japanese LIWC dictionary is available for academic researchers.

Constructing a Japanese version of the English LIWC dictionary is not easy. Linguistic distance (the closeness of language structure) is the farthest between English and Japanese (Chiswick and Miller, 2005). The complexity of the Japanese language comes from its unique writing system composed of three scripts: *hiragana* (the Japanese cursive syllabary), *katakana* (the square Japanese syllabary), and *kanji* (Chinese characters used in Japanese writing). In addition, the word classification system is based on three different origins: *wago* (or *Yamato kotoba*; native Japanese words), *kango* (Chinese-origin words), and *gairaigo* (words borrowed from foreign, mainly European, language), and their mixture. The multiple origins make the same *kanji* script have two or more pronunciations called *on-yomi* (based on *kango*) and *kun-yomi* (based on *wago*), which often have the same meaning. Moreover, the Japanese language has a rich variation of onomatopoeia that describe real sounds (*giongo*), animal and human sounds (*giseigo*), and conditions and states (*gitaigo*) in both *hiragana* and *katakana*. Consequently, the average vocabulary size for native Japanese speakers (undergraduates) is between 30,000–50,000 (Sato N. et al., 2017), whereas it is around 20,000 for native English speakers (Nation and Waring, 1997). In the process of developing the J-LIWC2015, these complicated issues must be considered; there is no shortcut.

**FIGURE 1:**
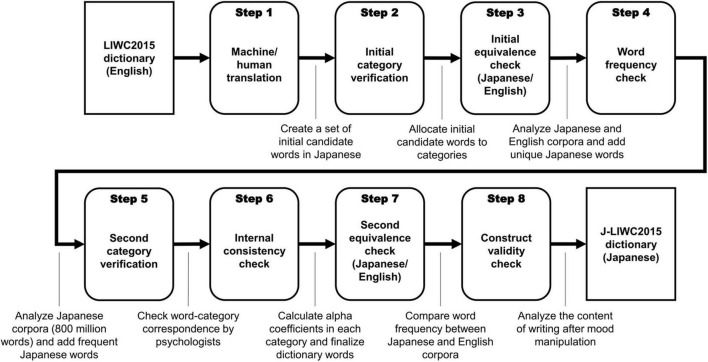
Procedure for the development of J-LIWC2015 dictionary.

In this study, we applied the standardized steps used to create translated LIWC dictionaries (Huang et al., 2012; Meier et al., 2019) with careful consideration of the Japanese language characteristics introduced above. The overall procedure involved eight steps (see Figure 1): First, we translated the target words in the LIWC2015 dictionary from English to Japanese (Step 1) and verified and adjusted the associations between the target words and the categories (Step 2), similar to Steps 1 and 2 of Matsuo et al. (2019). Then, we examined the word-category associations in large corpora and tested the equivalence between the LIWC2015 and J-LIWC2015 dictionaries (Step 3). We added high-frequency Japanese words to the dictionary and associated them with the categories (Step 4), followed by the fine-tuning of the category composition (Step 5). We then calculated the internal consistency of each category in large corpora (Step 6) and reexamined the correspondence between the English and Japanese versions of the dictionary in another dataset (Step 7). Finally, we conducted an online essay-writing experiment that manipulated participants’ moods to test the construct validity of each category (Step 8).

## Dictionary Development

### Step 1: Initial Translation

First, KS (computer scientist; third author of the present study), assisted by a computer science undergraduate student, used the online dictionary Weblio^1^ to directly translate all English target words in the LIWC2015 dictionary (hereafter referred to as “source-target words”) into Japanese. The source-target words with a wildcard were transformed into multiple English words using the online dictionary search tool OneLook^2^ before processing in Weblio. Every source-target word was translated into as many single or multiple Japanese words as possible. The Balanced Corpus of Contemporary Written Japanese (BCCWJ; 104 million words)^3^ and the Tsukuba Web Corpus (TWC; 1.1 billion words)^4^ were used to check the frequency of use of all translated words. Finally, a list of initial candidate words for J-LIWC2015 was created based on the translated words included in either corpus.

After the machine-translation process, the initial candidate words were manually screened by a group of reviewers, including TI (social psychologist; first author), KS, and five psychology students. All reviewers have either lived in English-speaking countries or are multilingual. The reviewers evaluated the machine-translated words and fixed or removed unnatural expressions. At this stage, the initial candidate words were standardized to written expressions used in daily life. If the source-target words represent abstract meanings or do not correspond directly to a single word in Japanese, the reviewers carefully chose translated expressions that reflect the original connotations. Some source-target words that were rarely used in Japanese, such as culture-specific words, abbreviations, and proper nouns (e.g., “Yiddish,” “DVR,” and “Zoloft”), were directly translated (“Yiddish” as it is in Japanese), remained (“DVR” as it is), or changed to words with a more general meaning (“Zoloft” was translated as “antidepressant” in Japanese) in this step. Abbreviated auxiliaries (e.g., “shouldn’t”) were not translated because no direct translation into single words is available in Japanese. For this reason, we were also unable to convert emoticons in the netspeak category (e.g., “:)”) and some source-target words in the informal and netspeak categories (e.g., “prob”).

Wildcards were added for three cases: (1) compound words that include *hiragana*, *katakana*, and/or *kanji* as a source of meaning, followed by other characters (e.g., a kanji “飲” means “drinking,” “飲食” means “drinking and eating,” and “飲酒” means “drinking alcohol,” therefore, “飲*” captures all expressions); (2) inflected forms of words (with modified endings) including verbs (conjugation) and adjectives (declension) (e.g., “教える” means “teach” as a verb, and it inflects as “教えて,” “教えた,” “教えろ,” and so forth according to the context; therefore, “教え*” captures all expressions); and (3) nominalized and adnominalized forms of words from adjectives (e.g., “小さい” means “small” as an adjective. “小ささ” means “smallness” as a noun, and “小さな” means “small” as adnominal; therefore, “小さ*” captures all expressions).^5^ LIWC prioritizes target words with wildcards; therefore, wildcards were only used if a character retained its meaning in modified forms of a word.^6^

Unlike English, spaces are not used to separate words when writing in Japanese (e.g., the sentence “彼は親切だ” (“he is kind”) is separated into four words “彼/は/親切/だ”). Thus, preprocessing texts by word segmentation is essential to determining word units in LIWC. We used the *de facto* standard tool for morphological analysis, MeCab Version 0.996^7^ (Kudo et al., 2004) with the IPA Dictionary (IPADIC) Version 2.7.0 (Asahara and Matsumoto, 2003), to segment text into words and attached a part of speech to each word.^8^ During the preprocessing, some single initial words were divided into two or more morphemes [e.g., “億万長者” (billionaire) is segmented as “億” (billion), “万” (ten thousand), and “長者” (rich person)]. We created a user dictionary for MeCab to handle these compound words as single-word units and confirmed that all initial candidate words were treated as single morphemes. The user dictionary is available on https://github.com/tasukuigarashi/j-liwc2015. Other preprocessing schemes, such as converting some double-byte characters (DBC) to single-byte characters (SBC) (e.g., symbols, numbers, and alphabet letters like “! A 1” convert into “!A1”), are introduced in a script that can be downloaded from the user dictionary link.

### Step 2: Initial Category Verification

The reviewers checked the correspondence between the initial candidate words translated into Japanese and the categories attached to their source-target words in English. The linguistic categories of J-LIWC2015 are slightly different from those of LIWC2015 because of the substantial differences in grammar between Japanese and English. Therefore, J-LIWC2015 does not include articles, prepositions, comparisons, and time orientation categories. Articles and prepositions are not used in Japanese, and the comparative degrees and tenses (time orientations) are expressed as adverbs and auxiliary verbs, respectively.^9^ If the same initial candidate words correspond to two or more source-target words that are linked to different categories, all relevant categories were attached to the single translated expressions (e.g., “利益” means both “benefit” [linked to the reward category] and “profit” [linked to the reward and work categories] in Japanese.^10^ Therefore, the translated word was linked to both reward and work categories). Categories linked to single source-target words were attached separately to two or more initial candidate words if single translated words could not cover all the categories (e.g., the source-target word “foundation*” is linked to the space and work categories, but no single Japanese word can represent both meanings. In this case, the translation is distinguished between “土台” for the former and “協会” for the latter category). The verbs category was attached to a few initial candidate words because many Japanese verbs are compound words of nouns and the auxiliary verb “する” (doing) and its inflections (e.g., “信頼” means “trust” as a noun and “信頼する” as a verb).

### Step 3: Initial Equivalence Check Between Japanese and English Dictionaries

After the initial translation and categorization, we compared the number of words allocated to each category in the English and Japanese versions of the dictionary. A research team in a Japanese linguistic technology company, specializing in natural language processing, compared the frequency of occurrence of the source-target words in English and the initial candidate words in Japanese (translated in Steps 1 and 2) in five large multilingual corpora: the English-Japanese Translation Alignment Data (Utiyama and Takahashi, 2003; 112,502 sentences), OpenSubtitles (2,082,927 sentences),^11^ Japanese-English Subtitle Corpus (JESC; Pryzant et al., 2018; 2,801,388 sentences), JEC Basic Sentence Data (5,304 sentences),^12^ and JParaCrawl (Morishita et al., 2020; 2,309,630 words). All of these include both English and Japanese texts for the same content.

If large discrepancies in the numbers of words in particular categories were found between the English and Japanese dictionaries, synonyms were searched through Japanese WordNet (Isahara et al., 2008) and added to the categories until the discrepancies were resolved. Many function words with inflections were added in this step. In the religion and netspeak categories, substantial discrepancies were observed between the two dictionaries. To fill this gap, the research team added new Japanese words that are not included in LIWC2015 but represent the meaning of the categories (e.g., “仏教” [Buddhism] and “2ch” [the largest internet forum in Japan like Reddit]) as closely as possible by searching relevant literature and the web.

### Step 4: Word Frequency Check

In this step, KS and SO (a computer science graduate student; the second author) checked the frequency of the words from large Japanese corpora in the current version of the dictionary, including BCCWJ, TWC, the National Institute for Japanese Language and Linguistics Web Japanese Corpus (NWJC; Asahara et al., 2014; 25 billion words), and the UniDic version of the Corpus of Spontaneous Japanese (CSJ; Watanabe et al., 2015; 7.5 million words from transcriptions of speech). Due to the low word frequency in the netspeak category in these corpora, random samples of Nicovideo Comment Etc. Data (DWANGO Co., Ltd, 2018; 17,349 videos) and Twitter data (scraped by the second and third authors: 569,760 words)^13^ were used to examine the frequency of the netspeak words included in the current version of the dictionary. Words with wildcards were extracted to multiple words by prefix searches in the corpora.

Word frequencies of each corpus were converted to normalized frequency scores based on frequencies per million words (PMW). Low-frequency words (PMW < 1 in all corpora) were removed from the dictionary. Words frequently used in the corpora but not listed in the translation-based dictionary were screened and added according to their relevance to each category. During the process of word addition, three new linguistic categories were created to include function words that could not be classified in the existing LIWC categories: case particles [particles placed after the nouns, such as “から” (from)], adjective verbs [a modified form of nouns that work like adjectives by adding a specific auxiliary verb “な” (-ous) at the end of the nouns, such as “贅沢な” (luxurious)^14^], and pronoun adjectival [adjectival words that are not categorized as either adjectives or adjectival verbs, such as “同じ” (equivalent)].

### Step 5: Second Category Verification

After compiling the dictionary based on word frequency, all words were sorted on a category-by-category basis. Then, a category evaluation team composed of TI and three graduate students (majoring in social psychology, developmental psychology, and cognitive science) thoroughly checked if each Japanese word accurately reflected the overall meaning of the category in the same way as in the English dictionary. Each team member evaluated the word-category correspondence individually. The team discussed the judgments and recategorized the words that were judged unfitting for a given category. Following the standard procedure to develop psychological assessment scales, the correspondence between a psychological construct behind each category and the words in that category were carefully examined. For example, all words in the affiliation and achievement categories were checked if they were generally fitted to one’s internal states and behavior derived from affiliation and achievement motivation theory (Atkinson, 1964; Hill, 1987). The team also amended minor errors in the dictionary, such as typos, in this step.

### Step 6: Internal Consistency Check

From a psychometric perspective, a reliable psychological measure needs to consistently represent the target psychological characteristics. According to Pennebaker et al. (2015), if an LIWC-like dictionary-based measure properly captures the psychological aspects of someone who produces a text, the text would contain multiple words associated with the same psychological category in the dictionary. For example, if a person writes a diary in Japanese about their positive experience, multiple words in the positive emotions category in J-LIWC2015, such as “楽し*” (fun) and “素晴らし*” (wonderful), would occur in the same text. The expressive pattern is regarded as internally consistent to the extent that a person’s positive emotions are consistently high across the text. Based on this idea, the internal consistency of each category in J-LIWC2015 was tested based on the procedure described below by Pennebaker et al. (2015).

In this step, TI, KS, and SO were engaged in the calculation of the internal consistency of each of the categories by using ten corpora, including more than 800 million words in total: Japanese novels and essays registered in Aozora Bunko,^15^ the minutes of plenary sessions and budget committees of the upper and lower houses of the National Diet of Japan,^16^ the Livedoor News Corpus,^17^ the Nagoya University Conversation Corpus (NUCC; Fujimura et al., 2012), the Nicovideo Comment Etc. Data, the Open 2 channel Dialog Corpus (Inaba, 2019), the Japanese subtitles of TED Talks,^18^ and the Twitter data used in Step 4. The Aozora Bunko and National Diet minutes corpora are provided by the Himawari full-text retrieval system.^19^ The total words and texts (a minimum unit of analysis that includes the same topic) and relative word counts, or the proportion of dictionary words used to the total number of words in each text, were computed in each corpus (see Supplementary Table 1).

Cronbach’s alpha coefficient was then calculated for each category in each corpus and averaged across the corpora (i.e., uncorrected alpha). The analysis regarded texts as responses, words as items, categories as factors, and the proportion of word use as item scores. One caveat is that Cronbach’s alpha coefficient calculation is not perfectly suitable when limited expressions are used in each free-descriptive text and when limited topics are covered in each corpus. In both cases, uncorrected alphas tend to be underestimated. The Spearman-Brown prediction formula was employed to adjust the bias under the assumption that the averaged alpha is observed in the ten corpora (i.e., corrected alpha) (Pennebaker et al., 2015).

The corrected alphas generally show high internal consistency, with some exceptions. The alphas were generally small in the personal pronoun category and its subcategories. The netspeak category also showed low internal consistency, probably due to the difficulty of allocating diverse expressions commonly used on the internet across different platforms and generations into a single category. In response to these results, very low and very high-frequency words were moved to different categories or removed from the dictionary to increase internal consistency. Although the alphas remained relatively low in the personal pronouns category even after the amendments, using pronouns in Japanese is not mandatory in a sentence and is often avoided to reduce redundancy. In particular, first- and second-person pronouns such as “私” (I) and “あなた” (you) are frequently dropped in Japanese in the context of conversations^20^ (Kashima and Kashima, 1998). Therefore, we conclude that having low alphas in these categories is not problematic in Japanese.

The final findings are presented in Table 1. The number of words removed in this step was very small (41 words; 0.4% of the dictionary words). After the amendments, we compiled the final version of the J-LIWC2015 dictionary, which includes 11,600 words and 69 categories (see Table 2).

**TABLE 1:** Internal consistency of each category.

Category	Uncorrected α	Corrected α	Category	Uncorrected α	Corrected α
Linguistic dimensions			Cognitive processes	0.478	0.901
Function words	0.511	0.913	Insight	0.353	0.845
Pronouns	0.195	0.708	Causation	0.176	0.681
Personal pronouns	0.045	0.321	Discrepancies	0.257	0.775
1st person singular	0.042	0.305	Tentative	0.317	0.823
1st person plural	0.029	0.229	Certainty	0.296	0.808
2nd person	0.044	0.316	Differentiation	0.359	0.849
3rd person singular	0.089	0.494	Perceptual processes	0.510	0.912
3rd person plural	0.039	0.287	See	0.477	0.901
Impersonal pronouns	0.204	0.720	Hear	0.425	0.881
Case particles*	0.369	0.854	Feel	0.310	0.818
Auxiliary verbs	0.359	0.848	Biological processes	0.572	0.930
Adverbs	0.434	0.885	Body	0.459	0.894
Conjunctions	0.242	0.762	Health	0.555	0.926
Negations	0.223	0.742	Sexual	0.302	0.812
Other grammar			Ingestion	0.490	0.906
Verbs	0.262	0.780	Drives	0.368	0.854
Interrogatives	0.261	0.779	Affiliation	0.287	0.801
Numbers	0.582	0.933	Achievement	0.313	0.820
Quantifiers	0.179	0.686	Power	0.401	0.870
Adjective verbs*	0.206	0.722	Reward	0.319	0.824
Pre-noun adjectival*	0.215	0.732	Risk	0.336	0.835
Psychological processes			Relativity	0.418	0.878
Affect	0.438	0.886	Motion	0.358	0.848
Positive emotions	0.355	0.847	Space	0.437	0.886
Negative emotions	0.428	0.882	Time	0.299	0.810
Anxiety	0.273	0.790	Personal concerns		
Anger	0.413	0.876	Work	0.541	0.922
Sadness	0.300	0.811	Leisure	0.352	0.844
Social processes	0.449	0.891	Home	0.398	0.869
Family	0.511	0.913	Money	0.617	0.941
Friends	0.137	0.614	Religion	0.292	0.805
Female references	0.385	0.862	Death	0.350	0.843
Male references	0.162	0.659	Informal language	0.396	0.868
			Swear words	0.222	0.741
			Netspeak	0.049	0.341
			Assent	0.102	0.531
			Non-fluencies	0.254	0.773
			Filler words	0.210	0.726

Internal consistency represents the mean values of alpha coefficients calculated at each of the ten corpora reported in Supplementary Table 1. Uncorrected α is Cronbach’s alpha coefficient averaged over the ten corpora. Corrected α is calculated based on the Spearman-Brown prediction formula. *Categories included only in J-LIWC2015 (not in LIWC2015).

**TABLE 2:** Categories of J-LIWC2015.

Category	Category (in Japanese)	Output label	Example	No. of words in category
Linguistic dimensions	言語次元			
Function words	機能語	Function	あいつ、非常に、ない	1808
Pronouns	代名詞	Pronoun	私、あなた、ここ	111
Personal pronouns	人称代名詞	Ppron	私、あなた、彼女	61
1st person singular	一人称単数	I	私、僕、おれ	13
1st person plural	一人称複数	We	我々、達、たち	3
2nd person	二人称	You	あなた、君、お前	16
3rd person singular	三人称単数	Shehe	彼女、彼、あいつ	8
3rd person plural	三人称複数	They	彼女ら、彼ら、奴等	8
Impersonal pronouns	不定代名詞	Ipron	ここ、之、全て	75
Case particles*	格助詞*	Casepart	とか、から、以来	187
Auxiliary verbs	助動詞	Auxverb	だ、できる、如し	110
Adverbs	副詞	Adverb	すっかり、非常に、目下	1268
Conjunctions	接続詞	Conj	すなわち、一方、及び	149
Negations	否定詞	Negate	ず、ない、未	44
Other grammar	その他の文法			
Verbs	動詞	Verb	わかる、行く、住む	2271
Interrogatives	疑問詞	Interrog	なぜ、どうして、何	18
Numbers	数詞	Number	ゼロ、百、率	52
Quantifiers	数量詞・助数詞	Quant	数多く、いろんな、バレル	261
Adjective verbs*	形容動詞	Adjverb	シンプル、大まか、平気	184
Pre-noun adjectival*	連体詞	Preadj	ほんの、そんな、如何なる	35
Psychological processes	心理的プロセス			
Affective processes	感情プロセス	Affect	おかしい、敏感、マジ	2067
Positive emotions	ポジティブ感情	Posemo	上品、善、最愛	941
Negative emotions	ネガティブ感情	Negemo	不信、いいかげん、いや	1075
Anxiety	不安	Anx	不吉、ナーバス、警鐘	157
Anger	怒り	Anger	不快、逆ギレ、ハラスメント	329
Sadness	悲しみ	Sad	不幸、悲観、自滅	168
Social processes	社会的（相互作用） プロセス	Social	迎える、語る、ふれあい	1027
Family	家族	Family	両親、いとこ、一家	122
Friends	友人	Friend	友達、盟友、相棒	92
Female references	女性	Female	ウーマン、乙女、ママ	137
Male references	男性	Male	おじさん、殿、婿	105
Cognitive Processes	認知プロセス	Cogproc	心がけ、図る、補う	1307
Insight	洞察	Insight	信念、注目、探索	384
Causation	原因	Cause	誘引、影響、動機	229
Discrepancies	不一致	Discrep	意外、ふつう、誤	107
Tentative	あいまいさ	Tentat	推定、気まぐれ、様々	281
Certainty	確かさ	Certain	常に、厳密、パーフェクト	247
Differentiation	差別化	Differ	識別、相違、対照的	137
Perceptual processes	知覚プロセス	Percept	五感、体験、味	970
See	視覚・知覚	See	一見、写す、白351	351
Hear	聴覚	Hear	歌唱、鳴る、静	205
Feel	感覚（触覚・味覚・嗅覚）	Feel	鋭利、感覚、うずうず	307
Biological processes	生物学的プロセス	Bio	遺伝、生態、成熟	956
Body	身体	Body	つま先、脳、眠い	286
Health	健康	Health	鎮痛、かゆい、しこり	384
Sexual	性	Sexual	ポルノ、欲情、変質者	118
Ingestion	摂取	Ingest	ビール、雑穀、ベジタリアン	225
Drives	動因	Drives	主権、威力、同胞	2083
Affiliation	つながり	Affiliation	身内、談笑、友情	418
Achievement	達成	Achieve	称賛、リーダー、誇る	546
Power	社会的地位・権力	Power	ランキング、当局、捕まる	959
Reward	報酬	Reward	発展、向上心、功績	237
Risk	リスク	Risk	事故、コスト、セキュリティ	252
Relativity	相対性	Relativ	原点、前後、無限	2342
Motion	動作	Motion	回す、握る、忍び足	844
Space	空間	Space	領域、距離、溝	760
Time	時間	Time	やっと、週末、最近	814
Personal concerns	個人的な事柄			
Work	仕事・学業	Work	転職、取り引き、幼稚園	837
Leisure	趣味・余暇	Leisure	歌う、園芸、小説	399
Home	家	Home	キッチン、枕、リフォーム	148
Money	金銭	Money	給料、負債、ディスカウント	317
Religion	宗教	Relig	密教、神主、供える	183
Death	死	Death	追悼、不滅、四十九日	89
Informal language	インフォーマル	Informal	何だ、あいつ、み	589
Swear words	罵倒	Swear	非国民、アホ、雑魚	56
Netspeak	ネットスラング	Netspeak	うｐ、なう、ぴえん	346
Assent	うなずき	Assent	そう、是非とも、おｋ	16
Non-fluencies	間投詞	Non-flu	あぁ、はて、へえ	121
Filler words	フィラー	Filler	うむ、さて、はは	25

Indents in the category row indicate hierarchical relationships among the categories. Output labels are used in the LIWC2015 software. *Categories included only in J-LIWC2015 (not in LIWC2015).

### Step 7: Second Equivalence Check Between Japanese and English Dictionaries

In this step, TI verified the equivalence between the final versions of J-LIWC2015 and LIWC2015. Two corpora were used for the analysis: TED Talks (subtitles available in both Japanese and English; 4,509 talks) and the Bible (the Old and New Testaments in the Colloquial Japanese Version and the Revised Standard Version in English; 1,372 chapters).^21^ Each category’s relative word counts were compared between Japanese and English texts in each corpus and the combined corpora. We computed Hedges’ *g* (i.e., unbiased Cohen’s *d* for two-sample *t*-test) and Pearson’s correlation coefficients (*r*) as equivalence indices of the Japanese and English versions of the dictionary. The similar the two versions, the larger *r*s and the smaller *g*s (*r*s are also expected to be positive). Supplementary Table 2 shows the results.

Overall, linguistic and non-linguistic categories did not show a large discrepancy between J-LIWC2015 and LIWC2015 in the combined corpora. In the verb category, a substantial discrepancy between J-LIWC2015 and LIWC2015 was observed. This finding is consistent with the dictionary editing polity introduced in Step 2. The space category showed a relatively larger gap than the other categories, probably because the English version includes frequent prepositions in the category, such as “on,” “off,” and “over,” that are part of idioms (e.g., “where are you off to?”) and do not directly correspond to single Japanese words. The frequency of words in the social category in J-LIWC2015 was also slightly lower than that in LIWC2015, probably because personal pronouns are allocated to this category in LIWC2015, whereas they are not frequently used in Japanese.

Relatively low correlation coefficients were also found in the subcategories of the informal language category because some proper nouns (e.g., “Ba’al-ha’nan” and “Josi’ah,” both of which are segmented by “’” (apostrophe) and “-” (hyphen) in the LIWC2015 software) in the Bible correspond to the words of the netspeak (e.g., “ha”) and assent (e.g., “ah”) categories in LIWC2015, but not in J-LIWC2015. Similarly, some proper nouns [e.g., “アッスリヤ” (Assyria)] correspond to the words of the non-fluencies category (e.g., “アッ*”) in J-LIWC2015, but not in LIWC2015. Note that this is not a specific issue of dictionary equivalence but a general issue of the word-count approach.

### Step 8: Construct Validity Check

At the final step of the dictionary construction, TI conducted an online experiment to confirm whether participants’ emotional state induced by an essay-writing task corresponded to the results of natural language processing by J-LIWC2015. In this step, we set the threshold for significance tests at *p* < 0.005 (Benjamin et al., 2018) to minimize Type I error.

#### Participants

Data from an online survey about daily experience and personality (Igarashi, 2019, Sample 2) was used in this study.^22^ A total of 522 Japanese crowdsourcing workers recruited *via* Lancers^23^ completed an online questionnaire. The questionnaire was created on Qualtrics and took 25.5 min (*SD* = 24.0) on average to complete. Each participant received 410 Japanese yen (approximately $4) for their remuneration. We excluded 22 answers from the data, including five participants who did not agree to write an essay, two participants whose essays were gibberish, and 15 participants who did not agree to use their answers for the study. Finally, we analyzed the data from 500 participants (305 women and 195 men, *M*_age_ = 37.9, *SD*_age_ = 9.85).^24^

#### Materials and Procedure

We induced participants’ emotional states using an essay-writing task (Oikawa and Oikawa, 2012). At the beginning of the survey, participants were asked if they would write an essay on one of the following topics: a positive experience (positive emotion condition; *n* = 164), a negative experience (negative emotion condition; *n* = 179), or an ordinary experience (control condition; *n* = 157) in recent days. Participants who agreed to write an essay were asked to provide a minimum of 200-characters in Japanese. They were asked to be expressive by referring to their emotional states while narrating their experience.

Upon completion, participants rated the pleasantness of the experience (1 item; “How was the experience for you?”) on a 6-point Likert scale (“1: very negative” to “6: very positive”). Participants were also asked to evaluate the impact of the experience (2 items; “I often remember the experience” and “the experience was important for me.”) on a 6-point Likert scale (“1: strongly disagree” to “6: strongly agree”). Responses to these scales were used for manipulation checks. Participants then reported their emotional states using the Japanese version of the Positive and Negative Affect Schedule (PANAS; Sato and Yasuda, 2001) (16 items; 6-point Likert scale), followed by the Big Five personality factors test (Namikawa et al., 2012) (29 items; 7-point Likert scale).

#### Content Analysis

**FIGURE 2:**
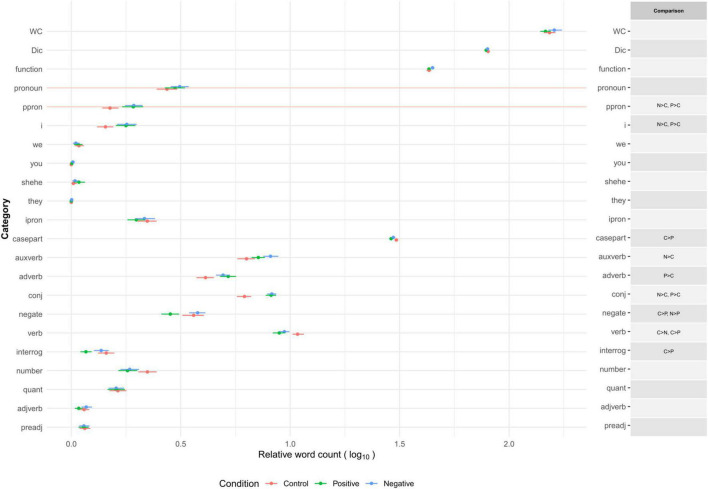
Word count and relative word count (% of total words) of linguistic categories across conditions in emotion manipulation task. Relative word count is the proportion of dictionary words used to the total number of words in each text. WC, (total) word count. Dic, % of dictionary words. Category names are shown as abbreviations reported in Table 2. Error bars represent 99.5% confidence intervals. Multiple comparison results are adjusted by Holm’s method (across WC, Dic, and all categories of J-LIWC2015 in Figures 2, 3) and shown here when adjusted as *p* < 0.005.

**FIGURE 3:**
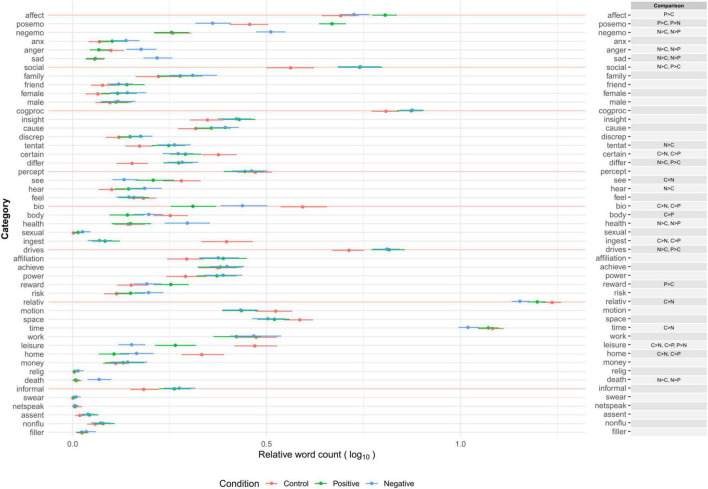
Relative word count (% of total words) of psychological categories across conditions in emotion manipulation task. Relative word count is the proportion of dictionary words used to the total number of words in each text. Category names are shown as abbreviations reported in Table 2. Error bars represent 99.5% confidence intervals. Multiple comparison results are adjusted by Holm’s method (across word count,% of dictionary words, and all categories of J-LIWC2015 in Figures 2, 3) and shown here when adjusted *p* < 0.005.

In this section, we examined whether there were substantial differences in word use in categories between two or more combinations of the three (positive emotion, negative emotion, and control) conditions. Mood manipulation was effective in inducing emotional states. The details of the statistical analysis (manipulation checks and multiple comparison tests) are reported in Section “Dictionary Development” in Supplementary Materials and Supplementary Table 3. On average, the dictionary words covered 78.6% (*SD* = 4.32) of the words that appeared in the essays. Figure 2 presents the descriptive information of the proportion of word occurrence (and word counts) in linguistic categories (function words and other grammars) in each condition. Figure 3 presents the average proportions of word occurrence in non-linguistic categories in each condition, followed by multiple comparison tests across the conditions.

Overall, the content of the essays corresponded to the induced emotional states. Participants in the control condition tended to write about non-social, leisure-related episodes at home, including something related to consumption (e.g., watching TV alone in the living room while eating snacks). In contrast, participants in the positive emotion condition tended to write about positive, social, drive-based, and leisure-related episodes (e.g., remembering the enjoyment of a visit to a museum with family). Participants in the negative emotion condition tended to write about negative (especially anger and sad), social, drive-based but death-related, and less healthy episodes (e.g., experiencing tiredness and hopelessness because of serious trouble with a colleague). The tendency of word usage in the linguistic and cognitive processes categories suggests that the episodes in these conditions were described as subjective and explanatory (i.e., frequent use of personal singular pronouns and conjunctions) with less specific and more contrasting expressions. Neither total word counts nor the proportion of dictionary words to overall word counts differed across conditions. The patterns indicate that the J-LIWC2015 dictionary has sufficient construct validity to capture various psychological characteristics reflected in verbal expressions observed in different emotional states.

## Discussion

The goal of the current study was to develop a Japanese version of the LIWC2015 dictionary. Through a series of systematic analyses, the J-LIWC2015 dictionary includes both the translated words from the original English version and frequent words adopted from large Japanese corpora with sufficient internal consistency across various linguistic and psychological categories. The overall equivalence between the J-LIWC2015 and LIWC2015 dictionaries was confirmed by comparing Japanese and English transcripts and texts. The construct validity of J-LIWC2015 was also endorsed by the content analysis of essay writings in the emotion induction task. The evidence suggests that J-LIWC2015 is a powerful research tool for social scientists to scrutinize the psychometric aspects embedded in Japanese texts.

J-LIWC2015 has great potential for future research in the era of large-scale social data analytics. Researchers can use the dictionary to analyze one’s psychological states from basic single-person pronouns to complicated psychological processes reflected in several forms of voluminous Japanese texts, such as posts on social networking sites, voice chat logs in online gaming, audio transcripts of interviews and videos, meeting minutes, and so forth. For example, Sasahara et al. (2021) analyzed Twitter posts by a beta version of J-LIWC2015 to examine the patterns of changes in consumers’ reactions toward the resell of essential goods (e.g., face masks and hand sanitizers) in Japan during the initial stage of the COVID-19 pandemic from March to August 2020. The longitudinal research showed that the peaks of drive-related category word use on Twitter corresponded to the propagation of news related to reselling, legal sanctions against face mask reselling for profits, and criticisms of those making profiting excessively from the crisis. These findings reflect the utility of validated text-based analytic tools for gauging psychological trends in public opinion on the internet.

The equivalence between the J-LIWC2015 and LIWC2015 dictionaries also facilitates a content analysis of texts written in the same context by speakers of different languages. For example, a cross-cultural study (Lopez et al., 2019) used LIWC2007 to analyze the content of Twitter posts in 2017 with the #MeToo hashtag (a social media hashtag used to confess and share experiences of workplace sexual harassment and other forms of victimization against women) in French and English. The findings revealed that French tweets included more aggressive (swear) expressions than English tweets. The same research design can now be applied to the comparative analysis of Japanese and English texts. However, researchers should keep in mind that it is not always appropriate to use the categories showing low corrected alphas (e.g., personal pronouns and netspeak categories) in cross-linguistic and cross-cultural studies. Meanwhile, recent research (Windsor et al., 2019) claims that the English-based LIWC2015 is universally applicable for analyzing texts (United Nations documents) machine-translated from several non-English source languages (not including Japanese) to English. Nonetheless, this approach may miss high-frequency words observed only in source languages. At least for the present moment, the coverage of this approach is limited to texts that do not include informal words or culture-specific expressions (see also Boot, 2021, for further discussion comparing machine-translated and dictionary-based approaches).

Although the current version of the J-LIWC2015 dictionary is adequately equivalent to the English version, one substantial difference between the two is that linguistic categories such as verbs are not actively assigned to the dictionary words in J-LIWC2015. It is often the case in Japanese that the same word can be assigned to a different part of speech according to the context of word use. More broadly, proper nouns and non-dictionary words might be mishandled in both J-LIWC2015 and LIWC2015, as the names in the Bible were regarded as part of the informal language in Step 6. All the issues mentioned above stem from the fact that the LIWC2015 software does not use context information and word co-occurrence patterns for word category classification.

If researchers require more detailed contextual information of Japanese text data, we suggest using MeCab with IPADIC in postprocessing in addition to preprocessing. Since its release in 2006, MeCab with IPADIC has been widely used for natural language processing in Japanese. At the preprocessing stage of the application of J-LIWC2015, MeCab/IPADIC is used for morphological analysis of Japanese text. In addition, the software can provide part of speech information for every word in the text estimated from word occurrence and conjunction costs (the likelihoods of two words to occur and linked calculated based on the part of speech information of frequent words in IPADIC). Upon necessity, researchers can add linguistic category information of the entire text by combining the result of MeCab/IPADIC analysis on the part of speech information with the output of the LIWC2015 software.

**FIGURE 4:**
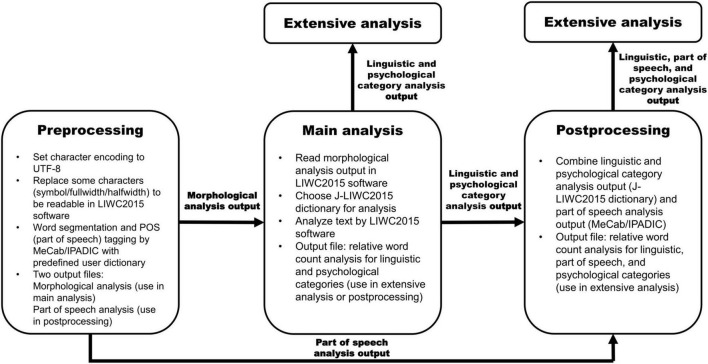
A general framework for quantitative language processing in Japanese by J-LIWC2015 dictionary. Sample scripts for preprocessing and postprocessing can be downloaded from https://github.com/tasukuigarashi/j-liwc2015.

Figure 4 introduces a general framework for the natural language processing of Japanese texts using the J-LIWC2015 dictionary, MeCab/IPADIC, and the LIWC2015 software. The framework contains the preprocessing, main analysis, and postprocessing stages. In the preprocessing stage, researchers convert Japanese texts to be readable in LIWC2015 software and conduct morphological and part of speech analyses in MeCab/IPADIC (researchers can also utilize part of speech information of each word for filtering non-dictionary proper nouns that may be mislabeled in J-LIWC2015, although this is beyond the scope of the current study). In the main analysis stage, researchers use the output of the morphological analysis in the LIWC2015 software for linguistic/psychological category assignments based on the J-LIWC2015 dictionary. Researchers can complete the analysis at this stage and use the output for extensive analysis. If they need more detailed part of speech information, they can proceed to the postprocessing stage and combine the output of linguistic/psychological categories (obtained at the main analysis stage) with the output of the part of speech information (obtained at the preprocessing stage) for extensive analysis. Postprocessing is optional and dependent on the research purpose. If researchers think integrating the outputs from the J-LIWC2015 dictionary and MeCab/IPADIC can get the best of both worlds, we recommend adopting this option. Supplementary Table 4 presents a case of analysis of postprocessing, showing that MeCab/IPADIC captures more words in the verb, adverb, and auxiliary verb categories than the J-LIWC2015 dictionary. Sample scripts for postprocessing can be downloaded from https://github.com/tasukuigarashi/j-liwc2015.

Efficient handling of different expressions is still an open issue in natural language processing. The diversity of Japanese scripts has led to the emergence of orthographical variants. For example, a combination of the scripts (e.g., “行う” (do) can be written as “行なう” and “おこなう”), use of dialects [e.g., “疲れた” (tired) is spoken as “こわい” in Hokkaido dialect], and the use of a traditional form of *kanji* script [e.g., “学校” (school) used to be written as “學校”] result in different expressions of the same words and meanings. We attempted to resolve this issue by including different expressions of the same words in the dictionary, but the current version of J-LIWC2015 does not systematically handle orthographical variants (e.g., the dictionary does not include traditional forms of kanji scripts). The conversion of the variants to standard expressions at the preprocessing stage will help increase the coverage rates of dictionary words.

The LIWC2015 software uses a word count approach based on a fixed lexicon without considering the context of word use. Meanwhile, recent research has reported a potential advantage of advanced machine-learning algorithms, such as the Bidirectional Encoder Representations and Transformations (BERT; Devlin et al., 2018) and the Generative Pre-trained Transformer 3 (GPT-3; Brown et al., 2020), over the traditional word count approach in natural language processing (Lake and Murphy, 2021). For example, when the effectiveness of linguistic/psychological category information assigned by BERT and LIWC2015 was compared in German to predict positive and negative communication behaviors in dyadic conversations, BERT was likely to outperform LIWC2015 (Biggiogera et al., 2021). There is no doubt that applying data-driven machine learning technology in this field has a promising future. However, one of the biggest drawbacks of the machine-learning approach is the lack of available training data; neural language models such as BERT need large-scale “labeled” data for training and fine-tuning, but such data are not often easily available in social science research. Furthermore, we should be careful not about performance but also the transparency and accountability specifications in data processing (Felzmann et al., 2019).

## Conclusion

To conclude, the J-LIWC2015 dictionary provides a better understanding of the psychometric properties of Japanese texts. The dictionary is expected to act as a significant bridge between quantitative and qualitative research in Japanese, which allows researchers to gain multifaceted and deep insights into the data. We hope that the dictionary will be used in a wide range of fields in Japanese text analysis and foster further innovations in social science.

## Author’s Note

A preliminary version of the manuscript appeared in the Telecommunications Advancement Foundation Research Report (Vol. 33, 2018, in Japanese) (https://www.taf.or.jp/files/items/1076/File/%E4%BA%94%E5%8D%81%E5%B5%90%E7%A5%90.pdf) and PsyArXiv (https://psyarxiv.com/5hq7d/). The J-LIWC2015 dictionary can be downloaded from http://www.liwc.net/dictionaries/ for the standard LIWC2015 academic license (i.e., non-commercial) users.

## Data Availability Statement

The raw data supporting the conclusions of this article will be made available by the authors, without undue reservation.

## Ethics Statement

The studies involving human participants were reviewed and approved by the Ethical Review Board of the Graduate School of Education and Human Development, Nagoya University. The patients/participants provided their written informed consent to participate in this study.

## Author Contributions

TI and KS designed the study and wrote the manuscript. TI conducted the experiment. TI, SO, and KS analyzed the data. All authors contributed to the article and approved the submitted version.

## Conflict of Interest

Pennebaker Conglomerates, Inc. owns all rights to the J-LIWC2015 dictionary. TI and KS obtained the correct permissions to develop the J-LIWC2015 dictionary as independent academic researchers. All royalties given to TI and KS for the commercial use of the J-LIWC2015 dictionary will be donated to Nagoya University and Tokyo Institute of Technology. The remaining author declares that the research was conducted in the absence of any commercial or financial relationships that could be construed as a potential conflict of interest.

## Publisher’s Note

All claims expressed in this article are solely those of the authors and do not necessarily represent those of their affiliated organizations, or those of the publisher, the editors and the reviewers. Any product that may be evaluated in this article, or claim that may be made by its manufacturer, is not guaranteed or endorsed by the publisher.

## Footnotes

1
https://ejje.weblio.jp/

2
https://www.onelook.com/

3
https://ccd.ninjal.ac.jp/bccwj/

4
https://tsukubawebcorpus.jp/

5 Adjectives in English correspond to adjectival verbs, adjectival nouns, and adnominals in Japanese.

6 Dictionaries used for translations in Step 1 are listed in section 1 of Supplementary Material.

7 Translation of the MeCab documentation to English is available at https://github.com/jordwest/mecab-docs-en.

8 MeCab can be used with other dictionaries, such as UniDic (https://ccd.ninjal.ac.jp/unidic/en/) and NEologd (Sato T. et al., 2017). However, the J-LIWC2015 dictionary is optimized for IPADIC. We cannot guarantee the compatibility of the findings if researchers use other dictionaries in MeCab for morphological analysis.

9 Although we could have added some adverbs and auxiliary verbs representing the comparative and tense categories in J-LIWC2015, we did not do so because there are no such grammatical categories in Japanese.

10 During the process of category assignments, we did not consider infrequent meanings of words. For example, “利益” also has a meaning of “blessing,” but we did not assign the word to the religion category because the use of the word in this context is rare.

11
http://www.opensubtitles.org/

12
http://nlp.ist.i.kyoto-u.ac.jp/EN/?JEC%20Basic%20Sentence%20Data

13
https://twitter.com

14 In MeCab, auxiliary verbs are divided into nouns and the auxiliary verb “な” (-ous). Considering that the English version of LIWC2015 does not include a category for nouns, we implemented the adjective verb category for the noun part of the words.

15
https://www.aozora.gr.jp/

16
https://kokkai.ndl.go.jp/

17
https://www.rondhuit.com/download.html#ldcc

18
https://www.ted.com/

19
https://www2.ninjal.ac.jp/lrc/index.php?himawari

20 Negative alpha coefficients in the personal pronouns category are found only in the conversation corpora (National Diet Minutes and NUCC).

21
http://www.babelbible.net/pdf/pdfmari.cgi?mode=right

22 In the survey, participants were asked to report demographic information (gender, age, academic background, and occupation), to write an essay after emotion induction manipulation, and to report emotional states, loneliness, and the Big Five personality factors. Igarashi (2019, Sample 2) used the demographic and psychological variables to verify factor structure and construct validity of a loneliness scale. The current study reports the result of essay content analysis based on J-LIWC2015, which is not included in Igarashi (2019, Sample 2).

23
https://www.lancers.jp

24 Sensitivity power analysis in G*Power3 (Faul et al., 2007) for one-way ANOVA (fixed effects, omnibus, three groups) shows that the effect size *(f*) of the current study (*N* = 500) is 0.178 with α (error probability) = 0.005 and β (power) = 0.80. This indicates that the current sample size is sufficient to detect small effects.
