# Supplementary material for: Development of the Japanese Version of the Linguistic Inquiry and Word Count Dictionary 2015
Source: Front Psychol. 2022 Mar 7;13:841534. doi: 10.3389/fpsyg.2022.841534 (PMC8940168; doi:10.3389/fpsyg.2022.841534)
Supplement: Supplementary file 1 [file Data_Sheet_1.docx]

**Supplementary Materials**

**Development of the Japanese Version of the Linguistic Inquiry and Word Count Dictionary 2015 (J-LIWC2015)**

**Tasuku Igarashi, Shimpei Okuda, and Kazutoshi Sasahara**

# Dictionaries used in Step 1 (Initial Translation)

For human translation, we referred to Longman Dictionary of Contemporary English (5th edition) (Pearson Education), Collins Cobuild English Dictionary for Advanced Learners (3^rd^ edition) (HarperCollins), Kenkyusha's New English-Japanese Dictionary (6^th^ edition) (Kenkyusha), Kenkyusha's New Japanese-English Dictionary (5^th^ edition) (Kenkyusha), Dictionary of English Collocations, the Kenkyusha (Kenkyusha), Kenkyusha's English-Japanese Dictionary for the General Reader (2^nd^ edition) (Kenkyusha), Kenkyusha's New College English-Japanese Dictionary (7^th^ edition) (Kenkyusha), Kenkyusha's New College Japanese-English Dictionary (5^th^ edition) (Kenkyusha), New Shogakukan Random House English-Japanese Dictionary (Shogakukan), Genius English-Japanese Dictionary (Taishukan Shoten), Genius Japanese-English Dictionary (2^nd^ edition) (Taishukan Shoten), Saito's Japanese-English dictionary (Nichigai Associates), Unno's Real English Dictionary V5 (Project Pothos), Kōjien (6^th^ edition) (Iwanami Shoten), Thesaurus (2^nd^ edition) (Taishukan Shoten), Lifescience (LSD Project), Eijiro (Electronic Dictionary Project), Kanjigen (5^th^ revised edition) (Gakken Plus), Urban Dictionary (<https://www.urbandictionary.com/>), and other internet resources.

# Results of Analysis of Variance in Step 8 (Construct Validity Check)

## Manipulation Check.

We examined whether the emotion manipulation worked properly. No substantial baseline difference in personality factors was found across the samples in the control, positive emotion, and negative emotion conditions. Multivariate analysis of variance (MANOVA) revealed that the variables varied across the control, positive emotion, and negative emotion conditions, Wilks’ Λ = 0.201, *F* (18, 978) = 65.2, *p* < .001, partial η^2^ = .545. Mean values of each variable in each condition and the results of analysis of variance (ANOVA) are reported in Supplementary Table 3. As expected, participants in the positive emotion condition rated their experience reported in the essay as more pleasant and positive than those in the control and negative emotion conditions. Participants in the negative emotion condition rated their experience as less pleasant and more negative than those in the control and positive emotion conditions. Participants in the control condition rated their experience as less impactful than those in the positive and negative emotion conditions. The positive affect score in the Positive and Negative Affect Schedule (PANAS) was the highest in the positive emotion condition, and the negative affect score in PANAS was the highest in the negative emotion condition. No substantial difference in personality factors was also observed across the conditions. One-sample *t*-tests on the impact score were also conducted to compare the mean values in each condition to the midpoint (7) of the scale (ranging from 2 to 12). The impact scores in the positive and negative emotion conditions were higher than the median, *t* (163) = 27.0, *p* < .001, *d* = 2.09, and *t* (178) = 14.0, *p* < .001, *d* = 1.05, whereas the score in the control condition was not, *t* (156) = 0.16, *p* = .872, *d* = 0.01. The findings indicate that the emotion manipulation was successful.

## Multiple Comparison Tests.

Figure 1 in the manuscript presents the descriptive information of the proportion of word occurrence (and word counts) in linguistic categories (function words and other grammars) in each condition. Figure 2 in the manuscript presents the average proportions of word occurrence in non-linguistic categories in each condition, each followed by multiple comparison tests across the conditions. All following results indicate differences between the conditions at the significant level of adjusted *p* < .005 (Holm’s method).

### Function words and other grammars.

First-person singular words (e.g., “私” (“I”)) and conjunctions (e.g., “また” (“or”)) appeared more in the positive and negative emotion conditions than in the control condition. Negates (e.g., “ない” (“none”)) were less frequently used in the positive emotion condition than in the negative emotion and control conditions. Verbs (e.g., “する” (“do”)) were more frequently used in the control condition than in the positive and negative emotion conditions.

### Affect.

Positive emotion words (e.g., “元気” (“fine”)) were more frequently used in the positive emotion category than in the other categories. In contrast, negative emotion words (e.g., “駄目” (“ruin”)), including words in the anger (e.g., “激怒” (“furious”)) and sad (e.g., “嘆く” (“lament”)) categories, were more frequently used in the negative emotion category than in the other categories.

### Social.

Words in the social category (e.g., “話す” (“talk”)) were more frequently used in the positive and negative emotion condition than in the control condition.

### Cognitive processes.

In the control condition, words in the certainty category (e.g., “必ず” (“sure”)) were more frequently used, and words in the difference category (e.g., “しかし” (“however”)) were less frequently used than in the other conditions.

### Biological processes.

The use of words in the biological process category was more frequent in the control condition than in the other conditions. Of those, health-related words (e.g., “腰痛” (“backache”)) were more frequently used in the negative emotion condition than in the other conditions. Ingestion-related words (e.g., “お菓子” (“sweets”)) were more frequently used in the control condition than in the other conditions.

### Drives.

Words in the drive category (e.g., “影響” (“effect”)) were more frequently used in the positive and negative emotion conditions than in the control condition.

### Personal concerns.

Leisure-related words (e.g., “遊ぶ” (“play”)) were frequently used the most in the control condition, followed by in the positive emotion condition and the negative emotion condition. Home-related words (e.g., “家” (“house”)) were more frequently used in the control condition than in the other conditions. Death-related words (e.g., “死*” (“die”)) were more frequently used in the negative emotion condition than in the other conditions.

### Perceptual process, relativity, and informal.

No category showed substantial differences in word use across two or more conditions.

# Sample scripts for preprocessing and postprocessing

The latest version of the preprocessing/postprocessing scripts (and related information) is available at <https://github.com/tasukuigarashi/j-liwc2015>. Figure 4 in the main article explains the contents of preprocessing and postprocessing.

Supplementary Table 1. Relative word count (% of total words) of each category in ten corpora.

| Category/Corpus | Aozora Bunko (novels) | Aozora Bunko (essays) | National Diet Minutes (plenary sessions) | National Diet Minutes (budget committee) | Livedoor News | NUCC | Nicovideo | Open 2channel | TED Talks | Twitter | Grand mean  (SD) |
| --- | --- | --- | --- | --- | --- | --- | --- | --- | --- | --- | --- |
| Unit of analysis (text) | Work | Work | Session | Session | News article | Dyadic conversation | Comments on video | Conversation with replies | Talk | Account |  |
| N. of texts | 3114 | 3722 | 6697 | 3786 | 7346 | 129 | 19041 | 181666 | 5156 | 2748 |  |
| N. of words | 42275370 | 11947794 | 74454270 | 149087438 | 4275203 | 1097307 | 502295443 | 24830799 | 10592456 | 568784 |  |
| Average word count per text | 13575.91 | 3210.05 | 11117.56 | 39378.62 | 581.98 | 8506.26 | 26379.68 | 136.68 | 2054.39 | 206.98 |  |
| Dictionary words (%) | 69.23 | 62.68 | 70.32 | 72.39 | 63.63 | 73.27 | 53.03 | 65.55 | 74.16 | 53.73 | 65.80 (7.64) |
| Linguistic dimensions |  |  |  |  |  |  |  |  |  |  |  |
| Function words | 41.85 | 36.73 | 37.89 | 42.71 | 33.65 | 38.96 | 25.45 | 38.46 | 43.98 | 27.69 | 36.74 (5.85) |
| Pronouns | 3.46 | 2.93 | 2.26 | 3.34 | 1.24 | 4.66 | 2.85 | 2.24 | 3.63 | 1.81 | 2.84 (0.94) |
| Personal pronouns | 1.40 | 1.03 | 0.78 | 0.87 | 0.39 | 1.09 | 0.68 | 0.88 | 1.30 | 0.79 | 0.92 (0.28) |
| 1st person singular | 0.62 | 0.58 | 0.19 | 0.49 | 0.20 | 0.52 | 0.11 | 0.21 | 0.88 | 0.42 | 0.42 (0.23) |
| 1st person plural | 0.09 | 0.12 | 0.03 | 0.06 | 0.09 | 0.06 | 0.02 | 0.03 | 0.46 | 0.06 | 0.10 (0.12) |
| 2nd person | 0.22 | 0.13 | 0.48 | 0.22 | 0.05 | 0.10 | 0.13 | 0.18 | 0.11 | 0.13 | 0.18 (0.11) |
| 3rd person singular | 0.31 | 0.10 | 0.00 | 0.00 | 0.09 | 0.04 | 0.13 | 0.17 | 0.18 | 0.05 | 0.11 (0.09) |
| 3rd person plural | 0.04 | 0.04 | 0.02 | 0.06 | 0.01 | 0.00 | 0.01 | 0.02 | 0.10 | 0.01 | 0.03 (0.03) |
| Impersonal pronouns | 2.16 | 1.99 | 1.50 | 2.40 | 0.90 | 3.58 | 2.19 | 1.42 | 2.47 | 1.07 | 1.97 (0.75) |
| Case particles | 27.98 | 25.30 | 27.45 | 28.30 | 24.83 | 18.19 | 12.66 | 22.65 | 29.40 | 17.08 | 23.38 (5.35) |
| Auxiliary verbs | 5.82 | 4.92 | 4.50 | 5.88 | 4.29 | 8.00 | 5.57 | 7.57 | 6.14 | 4.87 | 5.76 (1.17) |
| Adverbs | 3.63 | 2.69 | 2.70 | 4.00 | 2.33 | 5.50 | 2.31 | 3.39 | 3.12 | 2.53 | 3.22 (0.93) |
| Conjunctions | 5.72 | 4.98 | 5.05 | 6.94 | 5.61 | 6.79 | 3.72 | 6.90 | 6.25 | 4.36 | 5.63 (1.05) |
| Negations | 2.42 | 2.06 | 1.45 | 2.16 | 1.69 | 3.74 | 3.19 | 3.96 | 2.05 | 2.23 | 2.49 (0.81) |
| Other grammar |  |  |  |  |  |  |  |  |  |  |  |
| Verbs | 6.23 | 4.76 | 4.72 | 4.61 | 5.37 | 5.62 | 3.98 | 6.53 | 6.39 | 5.39 | 5.36 (0.81) |
| Interrogatives | 0.48 | 0.29 | 0.10 | 0.28 | 0.23 | 1.09 | 0.42 | 0.49 | 0.59 | 0.31 | 0.43 (0.26) |
| Numbers | 1.88 | 2.89 | 3.36 | 3.71 | 1.95 | 1.72 | 1.15 | 1.08 | 1.42 | 1.44 | 2.06 (0.89) |
| Quantifiers | 0.48 | 0.62 | 0.82 | 0.99 | 0.94 | 0.78 | 0.61 | 0.76 | 1.02 | 0.74 | 0.78 (0.17) |
| Adjective verbs | 0.13 | 0.17 | 0.35 | 0.27 | 0.32 | 0.09 | 0.17 | 0.27 | 0.31 | 0.19 | 0.23 (0.08) |
| Prenoun adjectival | 0.27 | 0.27 | 0.21 | 0.76 | 0.18 | 0.59 | 0.11 | 0.34 | 0.25 | 0.16 | 0.31 (0.19) |
| Psychological processes |  |  |  |  |  |  |  |  |  |  |  |
| Affect | 2.67 | 2.41 | 3.14 | 2.52 | 3.60 | 3.49 | 4.89 | 4.38 | 3.62 | 4.21 | 3.49 (0.79) |
| Positive emotions | 1.28 | 1.33 | 1.88 | 1.30 | 2.37 | 1.90 | 2.93 | 2.11 | 2.25 | 2.71 | 2.01 (0.55) |
| Negative emotions | 1.27 | 0.94 | 1.18 | 1.09 | 1.04 | 1.30 | 1.71 | 1.96 | 1.21 | 1.28 | 1.30 (0.29) |
| Anxiety | 0.21 | 0.13 | 0.23 | 0.17 | 0.19 | 0.11 | 0.12 | 0.21 | 0.23 | 0.16 | 0.18 (0.04) |
| Anger | 0.46 | 0.32 | 0.26 | 0.21 | 0.33 | 0.45 | 0.67 | 0.78 | 0.33 | 0.45 | 0.43 (0.17) |
| Sadness | 0.26 | 0.22 | 0.11 | 0.08 | 0.14 | 0.15 | 0.33 | 0.26 | 0.17 | 0.22 | 0.19 (0.07) |
| Social processes | 4.14 | 3.24 | 4.50 | 3.54 | 4.49 | 3.62 | 2.72 | 4.03 | 4.11 | 4.42 | 3.88 (0.56) |
| Family | 0.63 | 0.40 | 0.04 | 0.04 | 0.36 | 0.41 | 0.26 | 0.28 | 0.33 | 0.30 | 0.31 (0.16) |
| Friends | 0.34 | 0.21 | 0.52 | 0.22 | 0.33 | 0.28 | 0.25 | 0.39 | 0.24 | 0.49 | 0.33 (0.10) |
| Female references | 0.61 | 0.33 | 0.02 | 0.02 | 0.44 | 0.21 | 0.22 | 0.24 | 0.24 | 0.22 | 0.25 (0.17) |
| Male references | 0.73 | 0.35 | 0.02 | 0.01 | 0.29 | 0.23 | 0.40 | 0.32 | 0.30 | 0.33 | 0.30 (0.19) |
| Cognitive Processes | 4.15 | 4.63 | 6.96 | 8.13 | 5.38 | 6.73 | 3.49 | 7.42 | 7.50 | 5.06 | 5.95 (1.52) |
| Insight | 1.04 | 1.07 | 2.31 | 2.64 | 1.68 | 1.64 | 0.80 | 2.08 | 2.24 | 1.39 | 1.69 (0.59) |
| Causation | 0.86 | 1.36 | 1.58 | 1.70 | 1.38 | 0.98 | 0.58 | 1.19 | 1.96 | 0.93 | 1.25 (0.40) |
| Discrepancies | 0.29 | 0.27 | 0.86 | 1.06 | 0.41 | 0.39 | 0.48 | 0.67 | 0.42 | 0.53 | 0.54 (0.24) |
| Tentative | 0.90 | 0.74 | 0.88 | 1.21 | 0.79 | 1.14 | 0.50 | 1.35 | 1.29 | 0.86 | 0.96 (0.26) |
| Certainty | 0.58 | 0.75 | 1.19 | 1.17 | 0.72 | 0.88 | 0.56 | 0.86 | 1.25 | 0.72 | 0.87 (0.24) |
| Differentiation | 0.76 | 0.69 | 0.71 | 1.20 | 0.79 | 2.05 | 0.77 | 2.06 | 1.12 | 1.08 | 1.12 (0.50) |
| Perceptual processes | 2.46 | 1.90 | 0.58 | 0.75 | 2.43 | 1.73 | 2.06 | 1.69 | 2.23 | 1.81 | 1.76 (0.61) |
| See | 1.25 | 0.99 | 0.19 | 0.23 | 1.34 | 0.56 | 0.79 | 0.62 | 1.02 | 0.78 | 0.78 (0.37) |
| Hear | 0.64 | 0.44 | 0.26 | 0.40 | 0.55 | 0.67 | 0.81 | 0.53 | 0.68 | 0.50 | 0.55 (0.15) |
| Feel | 0.47 | 0.34 | 0.08 | 0.09 | 0.35 | 0.33 | 0.28 | 0.38 | 0.35 | 0.36 | 0.30 (0.12) |
| Biological processes | 1.80 | 1.24 | 0.43 | 0.32 | 1.18 | 1.13 | 1.11 | 1.26 | 1.79 | 1.47 | 1.17 (0.46) |
| Body | 1.22 | 0.63 | 0.08 | 0.07 | 0.44 | 0.37 | 0.60 | 0.49 | 0.72 | 0.57 | 0.52 (0.31) |
| Health | 0.29 | 0.34 | 0.26 | 0.16 | 0.31 | 0.22 | 0.19 | 0.36 | 0.76 | 0.37 | 0.33 (0.16) |
| Sexual | 0.02 | 0.03 | 0.00 | 0.00 | 0.06 | 0.01 | 0.17 | 0.14 | 0.06 | 0.13 | 0.06 (0.06) |
| Ingestion | 0.30 | 0.25 | 0.10 | 0.09 | 0.34 | 0.56 | 0.19 | 0.33 | 0.29 | 0.39 | 0.28 (0.13) |
| Drives | 3.33 | 3.62 | 8.83 | 6.37 | 5.22 | 2.81 | 3.41 | 4.81 | 5.69 | 4.60 | 4.87 (1.71) |
| Affiliation | 0.85 | 0.77 | 1.46 | 0.92 | 1.47 | 0.78 | 0.86 | 1.06 | 1.07 | 1.54 | 1.08 (0.29) |
| Achievement | 0.74 | 1.33 | 2.05 | 1.53 | 1.71 | 0.80 | 0.93 | 1.43 | 1.93 | 1.13 | 1.36 (0.44) |
| Power | 1.56 | 1.37 | 4.61 | 3.20 | 1.63 | 0.92 | 1.33 | 1.80 | 2.13 | 1.54 | 2.01 (1.04) |
| Reward | 0.35 | 0.40 | 1.02 | 0.69 | 0.80 | 0.31 | 0.49 | 0.66 | 0.95 | 0.57 | 0.62 (0.23) |
| Risk | 0.39 | 0.31 | 1.04 | 0.96 | 0.50 | 0.28 | 0.39 | 0.71 | 0.70 | 0.46 | 0.57 (0.25) |
| Relativity | 11.96 | 10.05 | 8.29 | 8.38 | 9.30 | 9.01 | 6.30 | 7.94 | 10.69 | 7.64 | 8.96 (1.55) |
| Motion | 2.24 | 1.50 | 1.05 | 1.25 | 1.45 | 1.78 | 1.15 | 1.58 | 1.95 | 1.35 | 1.53 (0.35) |
| Space | 3.19 | 2.47 | 2.66 | 2.51 | 2.58 | 2.52 | 1.52 | 2.29 | 3.31 | 2.02 | 2.51 (0.49) |
| Time | 7.38 | 6.62 | 4.92 | 5.01 | 5.74 | 5.54 | 3.93 | 4.52 | 5.89 | 4.77 | 5.43 (0.97) |
| Personal concerns |  |  |  |  |  |  |  |  |  |  |  |
| Work | 0.89 | 1.68 | 7.40 | 4.71 | 2.81 | 1.44 | 0.63 | 1.91 | 2.55 | 1.53 | 2.55 (1.95) |
| Leisure | 0.46 | 0.71 | 0.10 | 0.07 | 1.87 | 0.58 | 0.86 | 0.81 | 0.71 | 0.88 | 0.71 (0.48) |
| Home | 0.43 | 0.26 | 0.10 | 0.08 | 0.20 | 0.20 | 0.06 | 0.15 | 0.23 | 0.18 | 0.19 (0.10) |
| Money | 0.31 | 0.33 | 1.92 | 2.01 | 0.86 | 0.42 | 0.21 | 0.60 | 0.50 | 0.73 | 0.79 (0.62) |
| Religion | 0.13 | 0.13 | 0.03 | 0.02 | 0.07 | 0.04 | 0.28 | 0.08 | 0.13 | 0.13 | 0.11 (0.07) |
| Death | 0.21 | 0.15 | 0.07 | 0.03 | 0.06 | 0.04 | 0.24 | 0.16 | 0.13 | 0.09 | 0.12 (0.07) |
| Informal language | 1.77 | 0.98 | 0.27 | 0.87 | 0.93 | 13.40 | 9.09 | 3.01 | 0.88 | 2.36 | 3.36 (4.13) |
| Swear words | 0.05 | 0.02 | 0.00 | 0.00 | 0.02 | 0.03 | 0.25 | 0.22 | 0.01 | 0.08 | 0.07 (0.09) |
| Netspeak | 0.02 | 0.02 | 0.01 | 0.02 | 0.07 | 0.97 | 1.10 | 0.32 | 0.14 | 0.33 | 0.30 (0.39) |
| Assent | 0.22 | 0.10 | 0.02 | 0.10 | 0.10 | 4.54 | 0.45 | 0.42 | 0.13 | 0.19 | 0.63 (1.31) |
| Nonfluencies | 0.77 | 0.44 | 0.10 | 0.25 | 0.19 | 3.95 | 6.34 | 1.01 | 0.17 | 0.67 | 1.39 (1.97) |
| Filler words | 0.25 | 0.13 | 0.04 | 0.20 | 0.08 | 1.78 | 3.41 | 0.41 | 0.12 | 0.34 | 0.68 (1.03) |

*Note.* Relative word count is the proportion of dictionary word use to the total number of words in each text. Grand mean (SD) is the unweighted mean value (standard deviation) across the ten corpora.

Supplementary Table 2. Relative word count (% of total words) in J-LIWC2015 and LIWC2015.

|  | TED Talks (*N* = 4509) | | | |  | Bible (*N* = 1372) | | | |  | TED + Bible (*N* = 5887) | | | |
| --- | --- | --- | --- | --- | --- | --- | --- | --- | --- | --- | --- | --- | --- | --- |
|  | Mean | | Equivalence | |  | Mean | | Equivalence | |  | Grand mean | | Equivalence | |
| Category | J-LIWC 2015 | LIWC 2015 | *g* | *r* |  | J-LIWC 2015 | LIWC 2015 | *g* | *r* |  | J-LIWC 2015 | LIWC 2015 | *g* | *r* |
| Word count (mean) | 1988.96 | 1623.76 | 0.34 | 0.979 |  | 831.26 | 666.39 | 0.42 | 0.460 |  | 1718.87 | 1400.41 | 0.30 | 0.967 |
| Dictionary words (%) | 74.19 | 85.10 | -2.43 | 0.592 |  | 75.49 | 85.98 | -2.30 | 0.377 |  | 74.50 | 85.31 | -2.39 | 0.540 |
| Linguistic dimensions |  |  |  |  |  |  |  |  |  |  |  |  |  |  |
| Function words | 43.96 | 53.28 | -2.35 | 0.496 |  | 49.03 | 58.13 | -2.11 | 0.373 |  | 45.14 | 54.41 | -2.03 | 0.555 |
| Pronouns | 3.57 | 14.37 | -3.52 | 0.708 |  | 6.80 | 15.73 | -2.48 | 0.289 |  | 4.33 | 14.68 | -3.08 | 0.486 |
| Personal pronouns | 1.27 | 7.72 | -2.71 | 0.738 |  | 4.17 | 11.55 | -2.10 | 0.356 |  | 1.95 | 8.61 | -2.19 | 0.627 |
| 1st person singular | 0.86 | 2.19 | -0.78 | 0.786 |  | 0.20 | 2.43 | -1.09 | -0.063 |  | 0.71 | 2.25 | -0.85 | 0.567 |
| 1st person plural | 0.46 | 1.90 | -1.49 | 0.416 |  | 0.57 | 0.68 | -0.10 | 0.293 |  | 0.49 | 1.62 | -1.07 | 0.297 |
| 2nd person | 0.11 | 1.66 | -1.56 | 0.529 |  | 1.52 | 2.80 | -0.63 | 0.337 |  | 0.44 | 1.93 | -1.05 | 0.426 |
| 3rd person singular | 0.18 | 0.82 | -0.71 | 0.718 |  | 0.58 | 3.61 | -1.58 | 0.187 |  | 0.27 | 1.47 | -0.81 | 0.510 |
| 3rd person plural | 0.09 | 1.14 | -1.92 | 0.460 |  | 0.65 | 2.04 | -1.08 | 0.244 |  | 0.22 | 1.35 | -1.34 | 0.403 |
| Impersonal pronouns | 2.43 | 6.64 | -2.84 | 0.564 |  | 2.97 | 4.17 | -0.77 | 0.018 |  | 2.56 | 6.06 | -2.09 | 0.206 |
| Auxiliary verbs | 6.14 | 8.28 | -1.32 | 0.355 |  | 5.74 | 7.60 | -0.79 | 0.360 |  | 6.05 | 8.12 | -1.13 | 0.360 |
| Adverbs | 3.09 | 5.40 | -1.97 | 0.513 |  | 2.69 | 2.51 | 0.16 | 0.075 |  | 3.00 | 4.73 | -1.19 | 0.425 |
| Conjunctions | 6.23 | 7.16 | -0.81 | 0.230 |  | 5.98 | 8.19 | -1.02 | 0.297 |  | 6.17 | 7.40 | -0.83 | 0.232 |
| Negations | 2.05 | 1.20 | 1.36 | 0.442 |  | 1.88 | 1.39 | 0.40 | 0.300 |  | 2.01 | 1.25 | 0.95 | 0.351 |
| Other grammar |  |  |  |  |  |  |  |  |  |  |  |  |  |  |
| Verbs | 6.39 | 15.16 | -3.60 | 0.256 |  | 6.23 | 13.86 | -2.78 | 0.174 |  | 6.35 | 14.86 | -3.35 | 0.234 |
| Interrogatives | 0.59 | 1.84 | -2.37 | 0.503 |  | 0.28 | 2.10 | -2.35 | 0.181 |  | 0.52 | 1.90 | -2.28 | 0.306 |
| Numbers | 1.40 | 2.02 | -0.52 | 0.832 |  | 1.15 | 1.53 | -0.20 | 0.280 |  | 1.35 | 1.91 | -0.41 | 0.594 |
| Quantifiers | 1.01 | 2.42 | -2.05 | 0.407 |  | 0.58 | 1.50 | -1.32 | 0.247 |  | 0.91 | 2.21 | -1.72 | 0.456 |
| Psychological processes |  |  |  |  |  |  |  |  |  |  |  |  |  |  |
| Affective processes | 3.64 | 4.18 | -0.35 | 0.803 |  | 4.05 | 4.23 | -0.07 | 0.590 |  | 3.73 | 4.19 | -0.25 | 0.705 |
| Positive emotions | 2.24 | 2.73 | -0.45 | 0.734 |  | 2.51 | 2.28 | 0.12 | 0.534 |  | 2.30 | 2.62 | -0.24 | 0.607 |
| Negative emotions | 1.23 | 1.40 | -0.18 | 0.835 |  | 1.53 | 1.94 | -0.31 | 0.347 |  | 1.30 | 1.52 | -0.21 | 0.658 |
| Anxiety | 0.24 | 0.27 | -0.10 | 0.781 |  | 0.18 | 0.33 | -0.42 | 0.153 |  | 0.22 | 0.29 | -0.18 | 0.625 |
| Anger | 0.34 | 0.36 | -0.06 | 0.710 |  | 0.78 | 0.65 | 0.20 | 0.248 |  | 0.44 | 0.43 | 0.03 | 0.536 |
| Sadness | 0.18 | 0.27 | -0.33 | 0.706 |  | 0.29 | 0.37 | -0.13 | 0.093 |  | 0.20 | 0.29 | -0.23 | 0.371 |
| Social processes | 4.11 | 9.76 | -2.01 | 0.786 |  | 7.73 | 15.55 | -2.24 | 0.260 |  | 4.96 | 11.11 | -1.70 | 0.743 |
| Family | 0.34 | 0.28 | 0.11 | 0.853 |  | 0.81 | 0.97 | -0.14 | 0.503 |  | 0.45 | 0.44 | 0.01 | 0.659 |
| Friends | 0.24 | 0.16 | 0.30 | 0.406 |  | 0.18 | 0.12 | 0.18 | 0.077 |  | 0.23 | 0.15 | 0.27 | 0.309 |
| Female references | 0.25 | 0.52 | -0.33 | 0.879 |  | 0.34 | 0.72 | -0.32 | 0.246 |  | 0.27 | 0.57 | -0.32 | 0.646 |
| Male references | 0.30 | 0.76 | -0.53 | 0.697 |  | 1.14 | 4.89 | -1.67 | 0.277 |  | 0.49 | 1.72 | -0.67 | 0.587 |
| Cognitive Processes | 7.50 | 11.70 | -1.89 | 0.767 |  | 4.69 | 6.66 | -0.81 | 0.454 |  | 6.85 | 10.53 | -1.29 | 0.791 |
| Insight | 2.25 | 2.51 | -0.25 | 0.805 |  | 0.78 | 1.02 | -0.29 | 0.482 |  | 1.90 | 2.16 | -0.22 | 0.822 |
| Causation | 1.96 | 2.11 | -0.19 | 0.652 |  | 1.09 | 1.09 | -0.01 | 0.095 |  | 1.76 | 1.87 | -0.13 | 0.648 |
| Discrepancies | 0.41 | 1.41 | -1.90 | 0.456 |  | 0.32 | 0.54 | -0.45 | 0.262 |  | 0.39 | 1.21 | -1.40 | 0.410 |
| Tentative | 1.28 | 2.56 | -1.56 | 0.582 |  | 0.92 | 1.02 | -0.11 | 0.307 |  | 1.20 | 2.20 | -1.04 | 0.510 |
| Certainty | 1.25 | 1.40 | -0.27 | 0.492 |  | 1.03 | 1.27 | -0.31 | 0.254 |  | 1.20 | 1.37 | -0.28 | 0.406 |
| Differentiation | 1.11 | 3.16 | -2.69 | 0.517 |  | 1.09 | 2.48 | -1.04 | 0.282 |  | 1.11 | 3.00 | -2.00 | 0.383 |
| Perceptual processes | 2.24 | 2.64 | -0.27 | 0.820 |  | 1.96 | 2.79 | -0.64 | 0.309 |  | 2.17 | 2.67 | -0.35 | 0.714 |
| See | 1.03 | 1.18 | -0.15 | 0.847 |  | 0.61 | 0.75 | -0.23 | 0.278 |  | 0.93 | 1.08 | -0.16 | 0.792 |
| Hear | 0.67 | 0.80 | -0.14 | 0.831 |  | 0.87 | 1.35 | -0.54 | 0.260 |  | 0.72 | 0.93 | -0.23 | 0.697 |
| Feel | 0.35 | 0.45 | -0.23 | 0.721 |  | 0.42 | 0.54 | -0.24 | 0.164 |  | 0.37 | 0.47 | -0.23 | 0.577 |
| Biological processes | 1.82 | 2.14 | -0.17 | 0.927 |  | 1.47 | 2.11 | -0.53 | 0.275 |  | 1.74 | 2.13 | -0.22 | 0.856 |
| Body | 0.73 | 0.75 | -0.02 | 0.886 |  | 0.90 | 1.14 | -0.28 | 0.267 |  | 0.77 | 0.84 | -0.07 | 0.774 |
| Health | 0.78 | 0.87 | -0.07 | 0.910 |  | 0.22 | 0.38 | -0.40 | 0.132 |  | 0.65 | 0.75 | -0.09 | 0.889 |
| Sexual | 0.06 | 0.10 | -0.14 | 0.761 |  | 0.02 | 0.06 | -0.28 | 0.059 |  | 0.05 | 0.09 | -0.15 | 0.725 |
| Ingestion | 0.29 | 0.45 | -0.21 | 0.935 |  | 0.36 | 0.48 | -0.22 | 0.205 |  | 0.31 | 0.45 | -0.21 | 0.823 |
| Drives | 5.72 | 7.89 | -1.05 | 0.711 |  | 5.77 | 7.57 | -0.73 | 0.310 |  | 5.73 | 7.82 | -0.96 | 0.590 |
| Affiliation | 1.07 | 2.89 | -1.50 | 0.478 |  | 1.23 | 1.43 | -0.15 | 0.228 |  | 1.11 | 2.55 | -1.10 | 0.344 |
| Achievement | 1.93 | 1.54 | 0.48 | 0.703 |  | 0.92 | 0.76 | 0.19 | 0.217 |  | 1.69 | 1.36 | 0.37 | 0.653 |
| Power | 2.16 | 2.50 | -0.29 | 0.794 |  | 3.30 | 4.73 | -0.81 | 0.238 |  | 2.43 | 3.02 | -0.39 | 0.642 |
| Reward | 0.95 | 1.13 | -0.32 | 0.468 |  | 0.71 | 0.73 | -0.03 | 0.195 |  | 0.90 | 1.04 | -0.25 | 0.435 |
| Risk | 0.71 | 0.52 | 0.41 | 0.730 |  | 0.52 | 0.35 | 0.27 | 0.135 |  | 0.67 | 0.48 | 0.36 | 0.518 |
| Relativity | 10.68 | 13.86 | -1.24 | 0.724 |  | 9.61 | 11.50 | -0.67 | 0.316 |  | 10.43 | 13.31 | -1.05 | 0.629 |
| Motion | 1.95 | 2.07 | -0.13 | 0.632 |  | 2.00 | 2.22 | -0.23 | 0.164 |  | 1.97 | 2.10 | -0.15 | 0.502 |
| Space | 3.32 | 7.43 | -2.45 | 0.767 |  | 2.88 | 6.25 | -1.89 | 0.301 |  | 3.22 | 7.16 | -2.26 | 0.654 |
| Time | 5.86 | 4.54 | 0.80 | 0.663 |  | 5.34 | 3.15 | 1.22 | 0.254 |  | 5.74 | 4.21 | 0.88 | 0.554 |
| Personal concerns |  |  |  |  |  |  |  |  |  |  |  |  |  |  |
| Work | 2.55 | 2.48 | 0.05 | 0.894 |  | 0.66 | 0.69 | -0.04 | 0.336 |  | 2.11 | 2.06 | 0.03 | 0.897 |
| Leisure | 0.71 | 0.88 | -0.20 | 0.855 |  | 0.35 | 0.39 | -0.08 | 0.155 |  | 0.62 | 0.77 | -0.17 | 0.803 |
| Home | 0.23 | 0.29 | -0.16 | 0.806 |  | 0.37 | 0.47 | -0.17 | 0.254 |  | 0.27 | 0.33 | -0.16 | 0.589 |
| Money | 0.49 | 0.61 | -0.14 | 0.939 |  | 0.21 | 0.31 | -0.23 | 0.115 |  | 0.43 | 0.54 | -0.14 | 0.885 |
| Religion | 0.13 | 0.20 | -0.16 | 0.924 |  | 1.45 | 3.34 | -1.09 | 0.332 |  | 0.44 | 0.93 | -0.36 | 0.713 |
| Death | 0.14 | 0.20 | -0.19 | 0.879 |  | 0.26 | 0.40 | -0.33 | 0.206 |  | 0.17 | 0.25 | -0.22 | 0.668 |
| Informal language | 0.85 | 0.41 | 0.77 | 0.473 |  | 0.80 | 0.48 | 0.50 | 0.002 |  | 0.84 | 0.43 | 0.70 | 0.341 |
| Swear words | 0.01 | 0.03 | -0.27 | 0.268 |  | 0.00 | 0.02 | -0.37 | -0.027 |  | 0.01 | 0.03 | -0.29 | 0.227 |
| Netspeak | 0.13 | 0.08 | 0.21 | 0.067 |  | 0.01 | 0.26 | -0.70 | -0.032 |  | 0.11 | 0.12 | -0.04 | -0.004 |
| Assent | 0.12 | 0.13 | -0.05 | 0.408 |  | 0.06 | 0.16 | -0.28 | -0.046 |  | 0.11 | 0.14 | -0.13 | 0.211 |
| Nonfluencies | 0.16 | 0.17 | -0.03 | 0.318 |  | 0.42 | 0.18 | 0.52 | 0.060 |  | 0.22 | 0.17 | 0.18 | 0.150 |
| Filler words | 0.11 | 0.01 | 1.00 | 0.064 |  | 0.11 | - | - | - |  | - | - | - | - |

*Note.* Relative word count is the proportion of dictionary word use to the total number of words in each text. J-LIWC2015 and LIWC2105 were used to analyze Japanese and English texts, respectively. Grand mean is the unweighted mean value across the TED Talks and the Bible texts. No filler words were counted in the Bible texts in English thus no equivalence check and grand mean calculation was made in the category. *g* = Hedges’ g (unbiased Cohen’s *d* for two-sample *t*-test). *r* = Pearson’s correlation coefficient.

Supplementary Table 3. Mean values of manipulation check and personality variables in each condition.

|  | Episode pleasantness | Episode Impact | Positive affect | Negative affect | Extraversion | Conscientiousness | Neuroticism | Openness | Agreeableness |
| --- | --- | --- | --- | --- | --- | --- | --- | --- | --- |
|  | － | (α = .69) | (α = .88) | (α = .91) | (α = .87) | (α = .93) | (α = .88) | (α = .83) | (α = .76) |
| Control (C) (*N* = 157) | 3.83 | 7.03 | 20.20 | 19.90 | 19.50 | 27.90 | 25.60 | 25.50 | 23.20 |
|  | (1.24) | (2.47) | (7.30) | (8.92) | (6.42) | (7.79) | (5.73) | (6.43) | (5.83) |
| Positive (P) (*N* = 164) | 5.38 | 10.30 | 26.80 | 20.10 | 19.50 | 28.40 | 24.00 | 25.20 | 22.10 |
|  | (1.14) | (1.58) | (7.63) | (8.34) | (5.90) | (6.68) | (6.36) | (5.99) | (5.05) |
| Negative (N) (*N* = 179) | 1.61 | 9.20 | 17.80 | 28.30 | 18.80 | 27.10 | 25.30 | 24.40 | 22.90 |
|  | (0.94) | (2.10) | (7.11) | (9.37) | (6.35) | (7.21) | (5.54) | (6.16) | (5.69) |
| *F* (2, 497) | 503.2 | 104.9 | 68.0 | 49.5 | 0.70 | 1.62 | 3.40 | 1.54 | 1.87 |
| *p* | < .001 | < .001 | < .001 | < .001 | .495 | .200 | .034 | .215 | .155 |
| 99.5%CI (N-C) | [-2.55, -1.87] | [1.53, 2.81] | [-4.67, -0.14] | [5.63, 11.12] | [-2.63, 1.21] | [-3.05, 1.41] | [-2.15, 1.47] | [-3.04, 0.78] | [-2.02, 1.40] |
| 99.5%CI (P-C) | [1.21, 1.90] | [2.66, 3.96] | [4.29, 8.91] | [-2.57, 3.03] | [-1.99, 1.93] | [-1.70, 2.86] | [-3.46, 0.24] | [-2.24, 1.66] | [-2.89, 0.59] |
| Multiple comparison (Holm; *p* < .005) | P>C>N | P>N>C | P>C>N | N>C, N>P |  |  |  |  |  |

*Note*. Multiple comparison results were adjusted by Holm’s method (adjusted *p* < .005).

Supplementary Table 4. Relative word counts (% of total words) and correlations of linguistic categories in J-LIWC2015 and MeCab.

| Category | J-LIWC2015 | MeCab | *r* |
| --- | --- | --- | --- |
| Verbs | 6.35 | 14.93 | 0.485 |
| Adverbs | 3.00 | 1.62 | 0.715 |
| Prenoun adjectival | 0.25 | 1.45 | 0.354 |
| Auxiliary verbs | 6.05 | 11.31 | 0.382 |
| Case particles | 30.01 | 30.80 | 0.683 |
| Filler words | 0.11 | 0.02 | 0.435 |

*Note*. TED + Bible (*N* = 5887) Japanese corpus in Supplementary Table 2 was analyzed. *r* = Pearson’s correlation coefficient.
